# Successful Assessment and Management of Chemothorax: A Case Report and Literature Review

**DOI:** 10.7759/cureus.86595

**Published:** 2025-06-23

**Authors:** Hassan Edward Bakali, Batuhan Riza Ecer, Rza Mammadov, Tevfik Ilker Akcam

**Affiliations:** 1 Thoracic Surgery, Ege University Faculty of Medicine, Izmir, TUR

**Keywords:** catheter malposition, central venous catheter complications, chemotherapy, chemothorax, chest drainage, pleural effusion, venous port catheter

## Abstract

Chemothorax is a rare complication associated with central venous catheters, caused by the inadvertent infusion of chemotherapy into the pleural space due to catheter malposition. Malignant pleural effusion is a common suspect in oncologic patients; however, alternative causes, such as chemothorax, should also be considered. Few cases have been reported, emphasizing the need for careful catheter placement and early recognition of complications to prevent adverse outcomes. The risk of this complication underlines the need for meticulous catheter placement, routine radiologic verification, and prompt management to prevent serious outcomes in affected patients.

## Introduction

Pleural effusion is defined as the accumulation of fluid between the parietal and visceral pleura. It can be classified as either transudative or exudative [1]. For transudative pleural effusion, possible causes include congestive heart failure, nephrotic syndrome, cirrhosis, urinothorax, superior vena cava obstruction, and glomerulonephritis [1,2]. For exudative pleural effusion, potential causes include infections, malignancies such as lymphoma, metastatic disease or mesothelioma, pulmonary embolism, and gastrointestinal system diseases [2,3].

In patients with known malignancy presenting with pleural effusion, clinicians often initially assume it may be malignant pleural effusion (MPE), especially if it presents unilaterally. MPE indicates disseminated disease and affects approximately 15% of cancer patients, with metastatic breast and lung carcinomas being the most common causes [4,5].

Central venous port devices, first introduced in 1982, have seen increasing use, particularly in oncologic patients. The ideal placement for the catheter tip is in the distal superior vena cava. Complications associated with port systems are categorized into early (within 30 days of implantation) and late (beyond 30 days), with an occurrence rate of up to 7-27% [6]. The most common issues include infection and catheter-related thrombosis. Given the potential for major complications and the affordability of chest radiographs, routine postoperative chest imaging is recommended [6].

While MPE is a frequent concern in cancer patients, other rare complications related to chemotherapy port catheters should also be considered. One such rare complication is chemothorax, defined as the accumulation of chemotherapy drugs within the pleural space, often due to catheter malposition or leakage. Recognizing chemothorax is crucial, as its presentation can mimic MPE, potentially leading to misdiagnosis. We present a case of a patient with a recently placed chemotherapy port catheter who developed unilateral pleural effusion immediately following the first administration of chemotherapy, an extremely rare complication of port catheter placement.

## Case presentation

**Figure 1:**
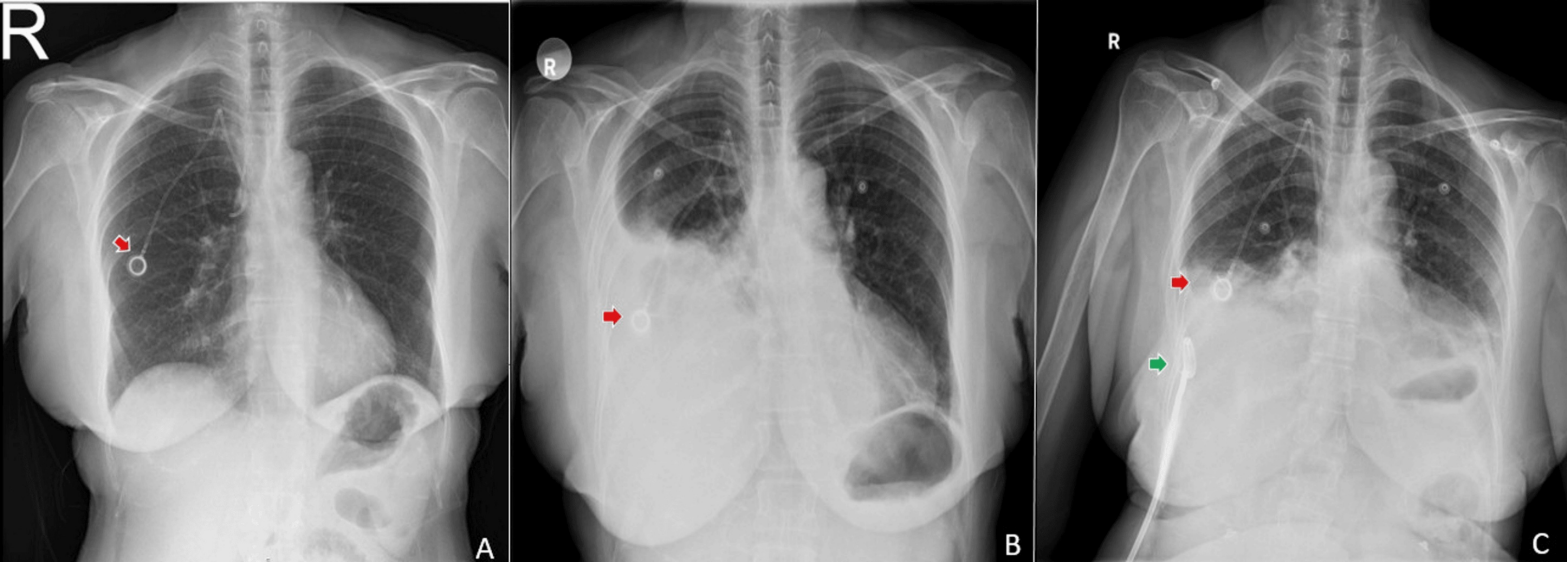
Preliminary chest radiographs of the patient. The port catheter is indicated by the red arrow, and the pigtail catheter is indicated by the green arrow. A: Control X-ray after port placement; B: Initial evaluation in the emergency room, showing right-sided pleural effusion; C: Control X-ray after pigtail catheter placement

A 64-year-old woman with a history of metastatic pancreatic cancer, who had previously received chemotherapy through a peripheral venous cannula, underwent surgical placement of a subclavian port catheter under ultrasound guidance for ongoing chemotherapy 10 days prior (Figure 1). She presented to our emergency department with complaints of cough and shortness of breath following her first administration of fluorouracil, leucovorin, irinotecan, and oxaliplatin (FOLFIRINOX) through the newly placed subclavian port catheter.

Upon initial evaluation, the patient’s vital signs were as follows: blood pressure of 171/91 mmHg, pulse rate of 122 beats per minute, body temperature of 36.3°C, and oxygen saturation of 90% while receiving 5 liters of oxygen per minute via nasal cannula. A posteroanterior chest X-ray revealed opacity in the lower zone of the right hemithorax, suggestive of pleural effusion (Figure 1). A 12F pigtail pleural drainage catheter was placed in the right hemithorax, and a total of 3,000 mL of clear serous fluid was gradually drained, which resulted in symptomatic relief (Figure 1).

Biochemical analysis of the pleural fluid sample showed a pH of 8, total protein below 2 g/L, albumin below 2 g/L, lactate dehydrogenase of 21 U/L, amylase below 3 U/L, and glucose level at 699 mg/dL (Table 1). The biochemical characteristics and saline-like appearance of the pleural fluid raised suspicion that the accumulation may have resulted from chemotherapy leaking into the pleural space through the subclavian port catheter.

**Table 1:** Biochemical analysis of the patient's pleural fluid, with reference ranges based on serum values

Test Name	Result	Unit	Reference range
Biological fluid pH	8		7.35 - 7.45
Total protein	<2	g/L	6.4 - 8.3
Albumin	<2	g/L	3.5 - 5.2
Lactate dehydrogenase	21	U/L	135 - 225
Amylase	<3	U/L	< 60
Glucose	699	mg/dL	60 - 140
Sodium	123	MEQ/L	136 - 145
Potassium	4	MEQ/L	3.5 - 5
Chloride	101	MEQ/L	96 - 110
Fluid cell count			
Leukocytes	0.06	10³/µL	4.5 - 11.0
Erythrocytes	0	10⁶/µL	3.8 - 5.2

**Figure 2:**
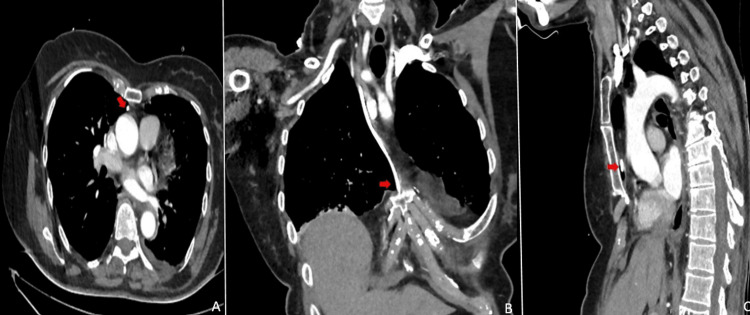
Patient's Contrast-enhanced computed tomography (CECT) Malpositioned port catheter, indicated by red arrows, displayed on multiple planes in chest CECT. A: axial, B: coronal, C: sagittal

**Figure 3:**
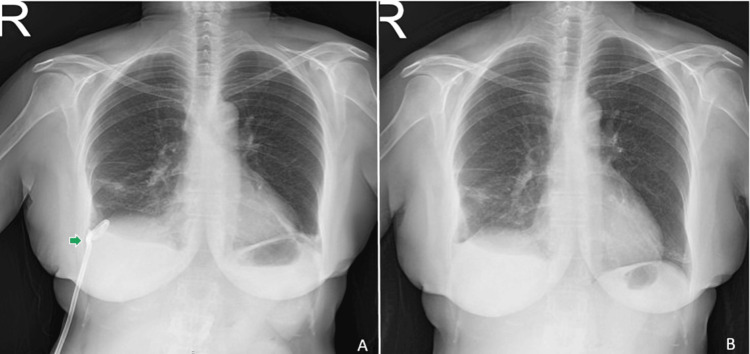
Patient's follow-up chest X-rays A: Control chest X-ray after removal of the port catheter. The pigtail catheter is highlighted by the green arrow. B: Control chest X-ray upon discharge after removal of the pigtail drainage catheter

Contrast-enhanced thoracic computed tomography (CECT) revealed that the port catheter, placed via the right subclavian vein, ended extravascularly in the mediastinum (Figure 2). The patient was evaluated by Cardiovascular Surgery due to the malpositioned catheter. She was admitted for observation, and the pleural drainage catheter was closely monitored. The port catheter was removed without complications, and the pleural drainage catheter was removed two days later, after a chest X-ray confirmed the absence of further drainage or fluid accumulation (Figure 3). The patient was discharged without further complications.

## Discussion

Under physiological conditions, the rate of pleural fluid formation and absorption is equal, maintaining a constant pleural fluid volume of approximately 10-20 ml [1]. In the differential diagnosis of pleural effusion, pathological conditions that can disrupt the balance between pleural fluid formation and absorption come to mind. In oncologic patients presenting with a unilateral pleural effusion, MPE comes to mind. MPE, which is a sign of end-stage disease, needs histopathologic verification, i.e., presence of malignant cells in the pleural fluid should be shown [3,4].

Central venous access catheters play a major role in patient care, especially in those with chronic illnesses. They can be used to withdraw blood or administer medications [6]. There are different types of venous access catheters, ranging from central lines to subcutaneously implanted access devices such as port catheters. Placement of port catheters, depending on the institution, can be performed by thoracic surgeons, cardiovascular surgeons, or, as is most common, interventional radiologists. Some studies have shown that catheters placed by interventional radiologists under ultrasound or even fluoroscopy have better outcomes with fewer complications compared to surgically placed ones [7].

Central venous access line placement may lead to various complications, including bleeding, air embolism, pneumothorax, catheter migration or malposition of the tip, cardiac perforation, and arrhythmias, among others [6,8]. The term "chemothorax" was first used by Kelly et al. [9] to describe a case in which chemotherapy was infused into the pleural cavity via a malpositioned port catheter. There are several cases in the literature where various substances or medications were erroneously administered through a malpositioned central access catheter [10-12].

Puttagunta et al. reported a case in which a patient received resuscitative fluids and propofol through a catheter that terminated intrapleurally, resulting in a case of pseudochylothorax [10]. Huang et al. [11] also reported a case where port catheter migration led to an intrapleural malposition. The patient developed a hydrothorax after fluid was administered through the catheter. Another case involved a malpositioned central venous catheter that led to an infusothorax of parenteral nutrition in a 16-year-old trauma patient [12].

Intrapleural chemotherapy, particularly agents such as bleomycin, has been employed for pleurodesis [13]. In cases like malignant pleural mesothelioma, intrapleural administration of cisplatin in conjunction with cytoreductive surgery has also been utilized for therapeutic purposes [14]. Only a few cases have been reported in which chemotherapeutic drugs were accidentally infused into the pleural cavity via a dislodged, malpositioned, or eroded central venous access port catheter (Table 2). These cases highlight the importance of imaging-guided catheter placement and meticulous checking of catheters before use. In both Kelly et al.'s case and ours, placement was performed under ultrasound guidance. Radiologic evaluations were also conducted, although these primarily focused on pneumothoraces.

**Table 2:** Published cases of chemothorax due to dislodged, malpositioned or eroded central venous access catheters N/A; Not available, VATS; Video-assisted thoracic surgery

Author	Year	Site of Catheter Placement	Placement Technique	Radiologic Check	Time Elapsed Between Placement and Complication	Infused Chemotherapy	Intervention
Pronchik et al. [15]	1998	Left subclavian vein	N/A	Chest X-ray	72 hours	Paclitaxel and carboplatin	Thoracentesis; catheter not removed
Kelly et al. [9]	2015	Right internal jugular vein	Ultrasound and fluoroscopy-guided	Chest X-ray	1 month	Docetaxel and cyclophosphamide	Right pleural drainage catheter; catheter removed
Panza et al. [16]	2022	Right subclavian vein	N/A	None	2 weeks	Cyclophosphamide and epirubicin	VATS pleural drainage and wash-out; catheter removed
Aguirre et al. [17]	2017	Right subclavian vein	Fluoroscopy-guided	Chest X-ray	N/A	Doxorubicin and cyclophosphamide	VATS pleural drainage and wash-out; catheter removed

There are no established guidelines for the management and treatment of these patients since chemothorax is a rare occurrence. Pronchik et al. [15] treated their patient with an initial thoracentesis. Antibiotics were also administered throughout the follow-up period since the patient refused repeated thoracentesis for the residual effusion. In both Kelly et al.'s case [9] and ours, a pleural drainage catheter (pigtail) was inserted.

At the other end of the spectrum, Panza et al. [16] and Aguirre et al. [17] managed their cases by performing a pleural washout and removing the dislodged central catheter via thoracoscopic surgery. They reported that thoracoscopic surgery allowed for safe removal of the catheter and effective irrigation of the pleural cavity with large amounts of saline.

Interestingly, only Pronchik et al. [15] chose not to remove the dislodged catheter, which may have contributed to the patient developing fever and septicemia. In the other three cases, as well as ours, the patients were discharged without further complications; however, Kelly et al.’s patient required multiple repeated thoracenteses during follow-up.

## Conclusions

This case highlights the importance of recognizing catheter malposition and chemotherapy accumulation in the pleural space. A multidisciplinary approach, including pleural drainage and thoracoscopy, is crucial. Clinicians should review unexpected findings thoroughly to enhance diagnostic accuracy. Catheter malposition can result from multiple factors; careful placement and anatomical knowledge are essential. Timely diagnosis and imaging-guided management can help prevent serious complications.

## References

[1] Light RW (2011). Pleural effusions. Med Clin North Am.

[2] Jany B, Welte T (2019). Pleural effusion in adults—etiology, diagnosis, and treatment. Dtsch Arztebl Int.

[3] Asciak R, Rahman NM (2018). Malignant pleural effusion: From diagnostics to therapeutics. Clin Chest Med.

[4] Gayen S (2022). Malignant pleural effusion: presentation, diagnosis, and management. Am J Med.

[5] Clive AO, Jones HE, Bhatnagar R, Preston NJ, Maskell N (2016). Interventions for the management of malignant pleural effusions: a network meta-analysis. Cochrane Database Syst Rev.

[6] Walser EM (2012). Venous access ports: indications, implantation technique, follow-up, and complications. Cardiovasc Intervent Radiol.

[7] Foley MJ (1995). Radiologic placement of long-term central venous peripheral access system ports (PAS Port): results in 150 patients. J Vasc Interv Radiol.

[8] Heberer M, Moser J, Dürig M, Harder F (1984). Prospective study of complications of central venous catheters [article in German]. Infusionsther Klin Ernahr.

[9] Kelly D, Geottman D, Sarodia B (2015). Chemothorax: a rare cause of a transudative pleural effusion. BMJ Case Rep.

[10] Puttagunta HK, Seneviratne C, Kupfer Y, Tessler S (2013). Pseudochylothorax and diaphragmatic weakness secondary to a misplaced central venous catheter. BMJ Case Rep.

[11] Huang CL, Lin PC, Lee JY, Chang YT (2012). Hydrothorax following delayed extravascular migration of a totally implantable venous access device in a child. J Pediatr Surg.

[12] Wildenauer R, Kobbe P, Waydhas C (2009). Bilateral hydrothorax and hydromediastinum after puncture of the right subclavian vein [article in German]. Unfallchirurg.

[13] Walker-Renard PB, Vaughan LM, Sahn SA (1994). Chemical pleurodesis for malignant pleural effusions. Ann Intern Med.

[14] Campany ME, Reck Dos Santos PA, Donato BB, Alwardt CM, Ernani V, D'Cunha J, Beamer SE (2023). Hyperthermic intrapleural chemotherapy: an update. J Thorac Dis.

[15] Pronchik DJ, Sexton J (1998). Emergency department presentation of an unusual pleural effusion. Am J Emerg Med.

[16] Panza T, Quercia R, Signore F (2022). Case report: Successful multimodal assessment and management of chemothorax. Front Surg.

[17] Aguirre VJ, Barnett D, Burdett N, Joshi R, Viana FF (2017). Video-assisted thoracoscopy in the management of intrapleural extravasation of cytotoxic chemotherapy. Thorac Cancer.

